# Multimodal In-training Examination in an Emergency Medicine Residency Training Program: A Longitudinal Observational Study

**DOI:** 10.3389/fmed.2022.840721

**Published:** 2022-03-09

**Authors:** Pin Liu, Shou-Yen Chen, Yu-Che Chang, Chip-Jin Ng, Chung-Hsien Chaou

**Affiliations:** ^1^Department of Emergency Medicine, Chang Gung Memorial Hospital, Lin-Kou Medical Center, Taoyuan, Taiwan; ^2^Department of Emergency Medicine, West Garden Hospital, Taipei, Taiwan; ^3^Graduate Institute of Clinical Medical Sciences, Division of Medical Education, College of Medicine, Chang Gung University, Taoyuan, Taiwan; ^4^Chang Gung, Medical Education Research Center, Taoyuan, Taiwan

**Keywords:** multimodal examination, high-fidelity simulation, in-training examination, emergency medicine, residency training

## Abstract

**Background:**

In-training examination (ITE) has been widely adopted as an assessment tool to measure residents' competency. We incorporated different formats of assessments into the emergency medicine (EM) residency training program to form a multimodal, multistation ITE. This study was conducted to examine the cost and effectiveness of its different testing formats.

**Methods:**

We conducted a longitudinal study in a tertiary teaching hospital in Taiwan. Nine EM residents were enrolled and followed for 4 years, and the biannual ITE scores were recorded and analyzed. Each ITE consisted of 8–10 stations and was categorized into four formats: multiple-choice question (MCQ), question and answer (QA), oral examination (OE), and high-fidelity simulation (HFS) formats. The learner satisfaction, validity, reliability, and costs were analyzed.

**Results:**

486 station scores were recorded during the 4 years. The numbers of MCQ, OE, QA, and HFS stations were 45 (9.26%), 90 (18.5%), 198 (40.7%), and 135 (27.8%), respectively. The overall Cronbach's alpha reached 0.968, indicating good overall internal consistency. The correlation with EM board examination was highest for HFS (ρ = 0.657). The average costs of an MCQ station, an OE station, and an HFS station were ~3, 14, and 21 times that of a QA station.

**Conclusions:**

Multi-dimensional assessment contributes to good reliability. HFS correlates best with the final training exam score but is also the most expensive format among ITEs. Increased testing domains with various formats improve ITE's overall reliability. Program directors must understand each test format's strengths and limitations to bring forth the best combination of exams under the local context.

## Background

During residency, periodic performance assessments, which facilitate the identification of strengths and weaknesses and help ensure program quality, are essential (1, 2). Various medical specialties have adopted In-training examinations (ITEs) as a powerful and multifunctional assessment tool to measure residents' competency (3–5). Written and oral examinations are the most common test formats adopted in ITEs. They have usually been applied to assess the degree of medical knowledge and clinical skills of the learners. However, oral and written test performance may not directly reflect residents' clinical experience and multitasking abilities, especially relevant for clinical competency in a busy and rushed clinical environment, such as the emergency department (E.D.) (6–8).

Simulations have been used in medical education since the 1960s (9). They have been integrated as a component of curricula emphasizing core competency and communication skills for emergency medicine (EM) residents (10, 11). The use of simulations in assessments has been extensively studied in anesthesiology (12, 13). Simulations can be used to evaluate residents' competency in differential diagnosis, resuscitation, and anesthesiology procedures (14, 15). Simulation-based assessment is also applied to EM residents, evaluating their milestones such as critical care and procedural skills (16). Simulation-based assessments can be formative or summative, and some studies have even supported the use of simulation-based assessments in board certification examinations (17, 18). High-fidelity simulation (HFS), which uses computer-controlled manikins, has been demonstrated to be realistic and effective in medical education (19, 20). The use of HFS in medical education has been reported to be associated with positive learning outcomes, both at the undergraduate and postgraduate levels (21–23). However, the high cost of HFS is a major obstacle to its implementation (24, 25).

There is a lack of literature to compare the different modes of assessments used in the ITEs. Understanding the nature of various assessment methods helps program directors gain a more holistic view of trainers' abilities. This longitudinal study examined the cost and effectiveness of the different testing formats within this multiformat biannual ITE.

## Methods

### Study Setting

This study was a retrospective analysis of educational data regularly collected between September 2015 and July 2019. The study site was the E.D. of a tertiary medical center in northern Taiwan with a 3,600-bed capacity and an annual E.D. census of 180,000 patient visits. The study site is one of the largest EM residency programs in Taiwan and accepts 7 to 10 new residents each year. This study was approved by a local institutional review board (I.R.B. No. 202000099B0) and was eligible for a waiver of informed consent.

EM residency training in Taiwan is a 3.5-year program. The program is designed and monitored by the Taiwan Society of Emergency Medicine (TSEM). The training sites are accredited annually by the TSEM according to the Residency Review Committee of the Taiwan Ministry of Health and Welfare guidelines. A complete description of the full residency training program is provided in Supplementary Table 1.

### Participants and Data Collection

The study enrolled a total of nine residents who were admitted to the EM residency program in 2015. Data from our biannual ITE and final EM board examination results were collected. The Taiwan EM board examination consists of single best answer MCQ test and oral examination stations. Each ITE round contained 8 to 10 stations concerning different topics and skill domains. Each station had one of four formats:

Multiple-choice question (MCQ) written tests: Each MCQ test contained 50 four-item, single-best-answer questions. The time limit was 50 min. The questions were all new, written for each examination by 5 to 10 EM faculty members.

Timed stations with questions and answers (QA): Each ITE had two to three QA stations. No examiners were required at the QA stations. The students rotated through the stations every 10 min. The questions were presented on a computer screen or paper. The topics suitable for this station format were electrocardiogram reading, image reading (radiograph, computed tomography, ultrasound), emergency dermatology, emergency ophthalmology, and emergency obstetrics/gynecology.

Oral examination (OE) stations: Each ITE had two to three OE stations. Within each OE station, one board-certified senior EM faculty member served as the examiner. The examiner examined the learner using prespecified test material and checklists. The OEs may contain several probing questions and were especially suitable for observing the clinical reasoning of residents.

Ultrasound or HFS: Each ITE included one ultrasound simulation and two HFS scenarios. The ultrasound simulation stations contained one rater, one standardized patient, and one teaching ultrasound machine equipped with phased array, curvilinear, and linear transducers. The test usually began with a scenario, and the examiner rated the residents using predefined point-of-care ultrasound checklists. Each HFS contained one rater, two standardized nurses, and one technical assistant. We used either a high-fidelity manikin or standardized patients with make-up. The topics were usually major EM topics such as pediatric emergency, emergency toxicology, medical emergency, and major trauma. The checklists included various competency domains such as communication skills, teamwork, leadership, and system-based practice routines.

Table 1 summarizes the number of stations per ITE, venue and faculty requirements, and topics and competencies tested by each format. Except for the MCQ stations, all stations were video-recorded for retrospective review or analysis. The costs of each format, including the expenses of the drafters of the test, the raters, the equipment, and the standardized patients or nurses, were also collected and analyzed.

**Table 1:** Characteristics of ITE formats.

	**Stations per ITE**	**Venue**	**Additional faculty or staff per station**	**Topics included**	**Clinical competencies**
Multiple choice question	1	Ordinary meeting room	1 administrative assistant	All contents related with Emergency Medicine	MK
Oral examinations	2–3	OSCE rooms	1 faculty as the rater	Pediatric emergency Emergency toxicology Medical emergency Disaster medicine Critical care medicine	PC / MK / ICS / SBP
Timed Q&A stations	2–3	OSCE rooms	None	ECG reading Image reading Dermatology (photos) Emergency ophthalmology Emergency OB-GYN	PC / MK
High fidelity simulations	3	Simulation center	1 faculty as the rater 2 standardized nurses 1 technical assistant	Pediatric emergency Emergency toxicology Medical emergency Major traumas	PC / MK / ICS / P / SBP

*Core competencies: PC, patient care; MK, medical knowledge; ICS, interpersonal and communication skills; P, professionalism; PBLI, practice-based learning and improvement; SBP, systems-based practice*.

### Statistical Analysis

Continuous variables are presented as mean (S.D.) and categorical variables as count and percentage. The reliability of the overall ITE and that of each format were calculated using Cronbach's alpha. The association between a resident's average ITE score and board examination results was evaluated using Pearson's correlation coefficient. Percentile scores of residents in this cohort vs. all residents in the program were evaluated to assess the validity of the training program using the ITE. All statistical analyses were performed using Microsoft Excel 2016 and SAS 9.4 (S.A.S. Institute Inc., Cary, NC).

## Results

A total of 486 station scores were recorded during the study period. The numbers of MCQ, OE, QA, and HFS stations were 45 (9.26%), 90 (18.5%), 198 (40.7%), and 135 (27.8%), respectively. The reliability of each format, measured using Cronbach's alpha, was lowest for MCQ (0.444) and highest for QA (0.935). The overall Cronbach's alpha reached 0.968, indicating good overall internal consistency (Table 2). The criterion validity, measured as the correlation with EM board examination results, was highest for HFS (ρ = 0.657), followed by MCQ (ρ = 0.634), QA (ρ = 0.571), and OE (ρ = 0.555) (Table 2).

**Table 2:** Quantitative ITE data: ITE scores, reliability of the format, resident satisfaction with the format, and correlation of scores with board examination results.

	**Number of station marks**	**Cronbach's alpha**	**Average satisfaction**	**Correlation with board**
Overall	486 (100%)	0.968	4.34 ± 0.78	0.620
Multiple choice question	45 (9.26%)	0.444	4.13 ± 0.77	0.634
Oral examinations	90 (18.5%)	0.846	4.42 ± 0.74	0.555
Timed Q&A stations	198 (40.7%)	0.935	4.30 ± 0.79	0.571
High fidelity simulations	135 (27.8%)	0.899	4.50 ± 0.76	0.657

**Figure 1:**
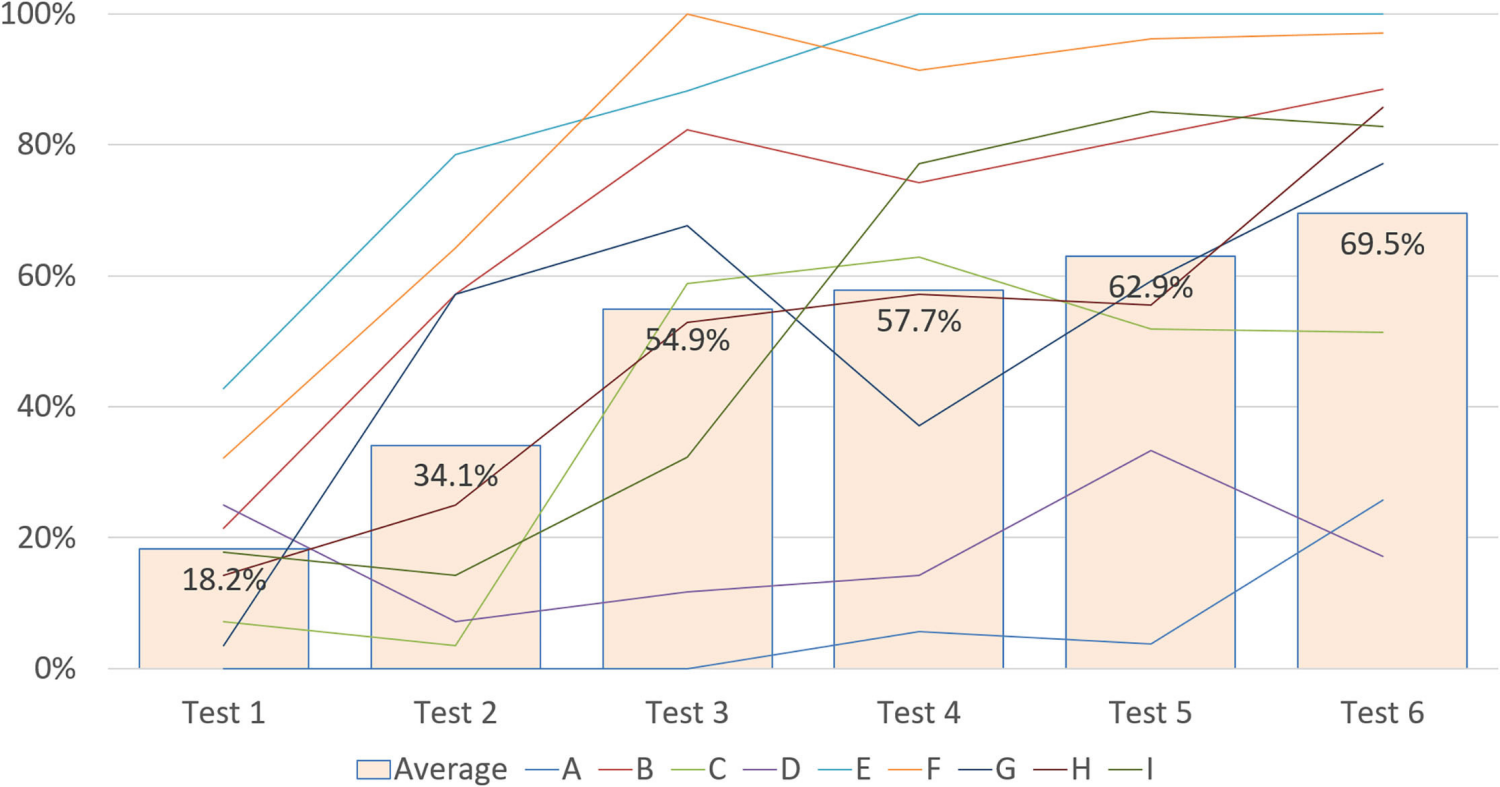
Progressions of individual and aggregate ITE percentile scores. Individual percentile scores are listed from lines A to I; the green bar represents the average percentile score of this cohort compared with all the residents in the program.

The progressions of resident ITE percentile scores are illustrated in Figure 1. Individual progressions are presented as colored lines, and the average percentile scores of this cohort compared with all the residents in the program are presented as green bars. As displayed in the figure, the average percentile score improved from 18.2% in the first year to 69.5% in the final year, indicating the effectiveness of the training program.

**Figure 2:**
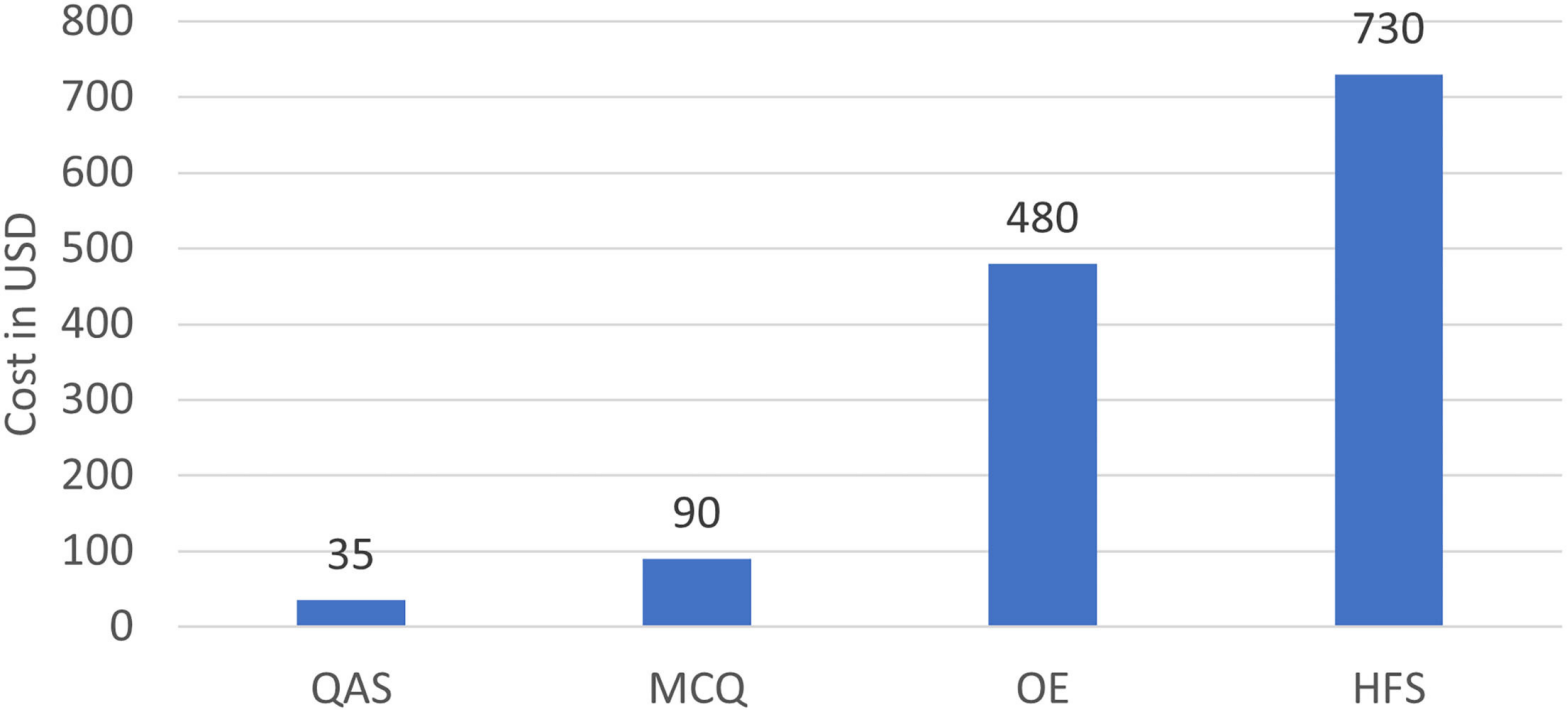
Average cost by format (per station, per day).

The average cost of the ITE stratified by format is displayed in Figure 2. Setting up QA stations for 1 day costs US$35, on average. The average costs of an MCQ station, an OE station, and an HFS station were ~3, 14, and 21 times that of a QA station. High learner satisfaction rates were reported from the OE and HFS stations, which both contained interactions with real people.

## Discussion

Our study examined the implementation of a multiformat ITE in an EM residency training program and demonstrated its validity and reliability. The average and individual ITE scores improved gradually with seniority. The ITE was also determined to exhibit good overall reliability, with HFS demonstrating the highest reliability. HFS was previously reported to have a reliability of 0.80 to 0.95, which is comparable to our study (26). In our ITE, the same rater was deployed to each HFS station. The rater used a structured checklist, thereby improving the objectivity of the scoring process and likely improving the consistency and reliability of the implemented HFS. Furthermore, HFS mimics clinical scenarios; the test content is close to clinical work and assesses residents' comprehensive competencies rather than rote medical knowledge. HFS tests different domains than MCQ, QA, and OE; hence, adding HFS to an ITE can increase the number of domains tested and improve the overall reliability of the ITE.

Medical education is currently oriented toward competency-based training. Training programs are challenged by the need to introduce appropriate and feasible assessment methods to evaluate the competency of residents. ITEs constitute a common tool used in residency training programs of multiple specialties. However, previous research has reported a poor correlation between ITEs and quantitative markers of clinical ability, such as patients per hour in EM or complication rates in anesthesiology (6, 27). Another study reported that clinical experience before an ITE was not correlated with examination scores (7). Traditional written and oral examinations used in ITEs may not accurately assess resident competency on their own. Simulation-based examinations and HFS have been demonstrated to accurately assess resident competencies across multiple domains (8, 28, 29). Integrating HFS into ITEs can improve the accuracy and efficiency of competency assessments and make them more comprehensive.

ITEs are used as summative assessments and as formative assessments for clinical teachers to know residents' deficiencies (30, 31). For specialty training, passing a board examination is the final outcome of the training program. The correlation between ITEs and board examinations has been studied in previous research, but the results were inconsistent (6, 32–34). Withiam-Leitch and Olawaiye reported that ITEs were weak assessment tools for predicting the probability of residents failing board examinations in obstetrics and gynecology in 2008 (32). Other studies have yielded different results and concluded that ITEs were suitable predictors of board examination scores in several specialties; improvement of ITE scores was also associated with an improvement in the pass rate (6, 33, 34). These diverse results may be attributed to the evolution of the ITE format. ITEs have become more similar to real board examinations, including written and oral examinations. HFS has long been added to the ITEs of our EM residency training program to establish a comprehensive and multifaceted assessment. HFS performance was found to have a higher correlation with board examination scores than performance on other test formats. Several studies have demonstrated that incorporating simulations into ITEs could improve the function of ITEs as a formative assessment and improve resident preparation for board examinations (35, 36). Furthermore, residents reported the highest satisfaction with HFS, and clinical teachers could evaluate learner competencies. Remedial teaching can be used for residents with lower ITE scores to improve their performance (37, 38).

Although HFS can increase the reliability and accuracy of ITEs, the cost of HFS is much higher than that of other test formats (25). Many educators have attempted to develop a low-cost HFS model or balance teaching efficacy and cost (39–41). However, HFS can compensate for the insufficiencies of other test formats; the benefit to learning outcomes is significant. The high cost of HFS engenders budgetary restrictions on how much it can be used in an ITE. Our study demonstrated that the use of HFS for 20 to 25% of an EM ITE can increase the reliability of the assessment and the ability of ITEs to predict board examination results without considerable extra cost. Determining the appropriate percentage of HFS use in ITEs of other specialties may warrant further research.

### Limitations

This study involved a single-center design; the results reflect the local situation. The generalizability of the results awaits confirmation from further studies. The detailed items of cost may also differ from country to country and from institution to institution. Furthermore, the study may have had selection bias and inadequate statistical power because of the small sample size. Our study also focused on the EM specialty and EM residents; further research is required to apply the results to other specialties.

## Conclusions

Multi-dimensional assessment contributes to good reliability. High-Fidelity simulation correlates best with final training exam score but is also the most expensive format among ITEs. Increased testing domains with various format improves ITE's overall reliability. Program directors must understand each test format's strengths and limitations to bring forth the best combination of exams under the local context.

## Author's Note

The authors of this article are board-certified EM physicians at Chang Gung Memorial Hospital in Taiwan. Our hospital is the largest EM resident training institution in Taiwan, and the authors are all members of the EM education committee.

## Data Availability Statement

The original contributions presented in the study are included in the article/Supplementary Material, further inquiries can be directed to the corresponding author.

## Ethics Statement

The studies involving human participants were reviewed and approved by Chang Gung Medical Foundation Institutional Review Board. Written informed consent for participation was not required for this study in accordance with the national legislation and the institutional requirements.

## Author Contributions

C-HC conceived, designed the study, performed data acquisition, conducted data analysis, and interpretation. Y-CC provided statistical expertise. S-YC wrote the manuscript draft. PL and C-JN made major revisions to the manuscript. All authors read and approved the final version of the manuscript.

## Funding

This study was funded by Chang-Gung Research Grant CDRPG3L0011. This research was supported by Chang Gung Memorial Hospital.

## Conflict of Interest

The authors declare that the research was conducted in the absence of any commercial or financial relationships that could be construed as a potential conflict of interest.

## Publisher's Note

All claims expressed in this article are solely those of the authors and do not necessarily represent those of their affiliated organizations, or those of the publisher, the editors and the reviewers. Any product that may be evaluated in this article, or claim that may be made by its manufacturer, is not guaranteed or endorsed by the publisher.

## Abbreviations

ED: emergency department
EM: emergency medicine
ITE: in-training examination
HFS: high-fidelity simulation
MCQ: multiple-choice question
OE: oral examination
QA: question and answer
TSEM: Taiwan Society of Emergency Medicine.
