# Supplementary material for: Multimodal In-training Examination in an Emergency Medicine Residency Training Program: A Longitudinal Observational Study
Source: Front Med (Lausanne). 2022 Mar 9;9:840721. doi: 10.3389/fmed.2022.840721 (PMC8959571; doi:10.3389/fmed.2022.840721)
Supplement: Supplementary file 1 [file Table_1.DOCX]

| **Year** | **Course** | **Months** |
| --- | --- | --- |
| 1 | Adult Emergency Medicine | 6 |
|  | General Pediatrics | 1 |
|  | General Internal Medicine | 1 |
|  | General Obstetrics and Gynecology | 1 |
|  | General Otorhinolaryngology | 1 |
|  | General Ophthalmology | 1 |
|  | General Surgery | 1 |
| 2 | Adult Emergency Medicine | 5 |
|  | Neurology | 1 |
|  | General Pediatrics | 1 |
|  | General Surgery | 1 |
|  | Disaster Medicine | 1 |
|  | Emergency Radiology | 1 |
|  | Critical Care Medicine | 2 |
| 3 | Adult Emergency Medicine | 5 |
|  | Critical Care Medicine | 2 |
|  | Emergency Medical Service | 1 |
|  | Pediatric Emergency | 1 |
|  | Emergency Psychiatrics | 1 |
|  | Emergency Toxicology | 1 |
|  | Emergency Ultrasound | 1 |
| 4 | Adult Emergency Medicine | 3 |
|  | Pediatric Emergency | 1 |
|  | Optional course | 2 |

**Emergency Medicine Residency Training Curriculum**

Reference: https://www.tma.tw/files/FilesDown/Cstandard/%E6%80%A5%E8%A8%BA%E9%86%AB%E5%AD%B8%E7%A7%91.pdf
